# Multiplexing 3D Natural Scaffolds to Optimize the Repair and Regeneration of Chronic Diabetic Wounds

**DOI:** 10.3390/gels11060430

**Published:** 2025-06-03

**Authors:** Cezara-Anca-Denisa Moldovan, Alex-Adrian Salagean, Mark Slevin

**Affiliations:** 1 Doctoral School of Medicine and Pharmacy, George Emil Palade University of Medicine, Pharmacy, Science, and Technology of Târgu Mureș, 540142 Târgu Mureș, Romania; moldovan.cezara@yahoo.com; 2 Department of Histology, George Emil Palade University of Medicine, Pharmacy, Science, and Technology of Târgu Mureș, 540142 Târgu Mureș, Romania; alex-adrian.salagean@umfst.ro; 3 Center for Advanced Medical and Pharmaceutical Research, George Emil Palade University of Medicine, Pharmacy, Science, and Technology of Târgu Mureș, 540142 Târgu Mureș, Romania

**Keywords:** diabetic foot ulcer, wound healing, 3D scaffolds, drug-delivery

## Abstract

Diabetic foot ulcers (DFU) represent a major complication of diabetes mellitus, affecting millions of patients worldwide and leading to high morbidity and amputation risks. The impaired healing process in DFU is driven by vascular insufficiency, neuropathy, chronic inflammation, and infections. Conventional treatments, including blood sugar control, wound debridement, and standard dressings, have shown limited efficacy in achieving complete healing. Recent advancements have introduced novel therapeutic approaches such as stem cell therapy, exosome-based treatments, and bioengineered scaffolds to accelerate wound healing and tissue regeneration. Mesenchymal stem cells (MSCs), particularly adipose-derived stem cells (ASCs), exhibit anti-inflammatory, pro-angiogenic, and immunomodulatory properties, enhancing wound repair. Additionally, exosomes derived from ASCs have demonstrated the ability to promote fibroblast proliferation, regulate inflammation, and stimulate angiogenesis. The integration of bioengineered scaffolds, including hydrogels, hyaluronic acid (HA), or micro-fragmented adipose tissue (MFAT), offers improved drug delivery mechanisms and a controlled healing environment. These scaffolds have been successfully utilized to deliver stem cells, growth factors, antioxidants, anti-glycation end products, anti-inflammatory and anti-diabetic drugs, or antimicrobial agents, further improving DFU outcomes. This review highlights the potential of combining novel 3D scaffolds with anti-diabetic drugs to enhance DFU treatment, reduce amputation rates, and improve patients’ quality of life. While promising, further clinical research is required to validate these emerging therapies and optimize their clinical application.

## 1. Introduction and Background

Diabetes Mellitus is one of the most prevalent chronic diseases across the world. It is considered to be a collection of metabolic disorders due to the multiple macrovascular and microvascular complications that co-exist with it [1]. More than 550 million people worldwide have diabetes, with a predicted increase in the upcoming years [2].

One of the main complications of DM is the diabetic foot ulcer (DFU), which leads to poor outcomes for many patients, increasing morbidity and mortality. It was reported that approximately 20–30% of patients with diabetes developed DFU, while amputation is required in 20% of these cases [3]. Approximately 18.6 million patients with diabetes develop DFU per year [2].

The pathophysiology behind DFU is primarily associated with neuropathy, the lack of sensation in the foot, which predisposes patients to trauma and ulceration. In addition, the metabolic changes due to hyperglycemia lead to vascular insufficiency and inflammation secondary to infection [4]. DFU is a difficult-to-manage entity that significantly affects the patient’s quality of life due to impaired healing.

## 2. Mechanisms Behind Impaired Wound Healing in DFU

The formation of DFU begins with arterial occlusion and lower limb ischemia due to atherosclerosis. Several factors, such as endothelial cell injury, vascular smooth muscle (VSMC) dysfunction, and a high inflammatory state, promote atherosclerosis. In addition, the hyperglycemic status and insulin resistance, along with activated advanced glycation end products (AGEs), lead to peripheral angiopathy, which is considered the initial cause of DFU and the main factor predicting amputation [5].

Diabetic neuropathy also significantly contributes to the development of DFU. Besides hyperglycemia, other major pathways like AGEs, the polyol pathway, hexosamine flux, poly-ADP ribose polymerase (PARP), and cyclooxygenase-2 (COX-2) reportedly contribute to the progression of neuropathy [6]. The primary consequence of neuropathy is loss of sensation in the foot. This facilitates progressive worsening of trauma to the lower extremities, as the initial insult is subjected to stress and plantar pressure due to walking and body weight. Also, autonomic neuropathy leads to dryness and keratinization of the foot, which also contribute to non-healing [7].

The most frequent complication of DFU is infection. These infections are very difficult to treat, making the treatment of the ulcer a challenge. The most common microorganisms involved in DFU are Gram-positive (*S. aureus*, *Streptococcus* spp., *Enterococcus* spp.) and Gram-negative organisms (*Pseudomonas* spp., *E. coli*, *Klebsiella* spp., *Proteus* spp.) [8].

**Figure 1:**
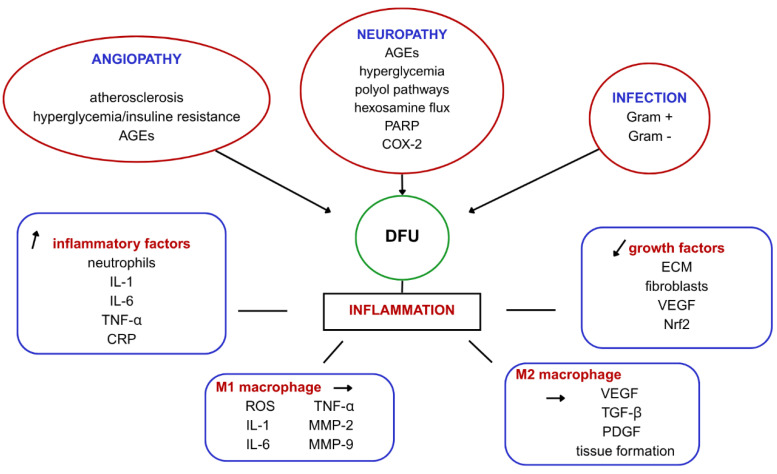
The figure illustrates the pathophysiological mechanisms involved in the development and progression of diabetic foot ulcers (DFUs), highlighting key contributing factors such as angiopathy, neuropathy, and infection. Angiopathy, including atherosclerosis and the accumulation of advanced glycation end-products (AGEs), impairs blood flow and tissue healing. Neuropathy, driven by AGEs, hyperglycemia, and the activation of various metabolic pathways, leads to nerve damage and loss of protective sensation. Infection, involving both Gram-positive and Gram-negative bacteria, exacerbates inflammation and delays healing. Inflammation, the central pathological process in DFUs, is driven by pro-inflammatory factors like neutrophils, IL-1, IL-6, TNF-α, and CRP, while M1 macrophages release factors that contribute to tissue damage and chronic inflammation. Impaired healing occurs due to reduced growth factors such as ECM fibroblasts, VEGF, and Nrf2, which delay tissue repair, while M2 macrophages, though promoting healing, are often suppressed in DFUs, further complicating the wound healing process.

Usually, wound healing undergoes four stages: hemostasis, inflammation, proliferation, and remodeling. DFU fails to progress through these stages and, therefore, remains in an inflammatory state for a longer period of time [9]. This is due to the imbalance between high inflammatory factors and the downregulation of growth factors in patients with diabetes. Insufficient re-epithelialization appears in association with impaired extracellular matrix (ECM) production and fibroblasts [10]. Also, because of the chronic inflammatory state, neutrophils and macrophages characterize the wound bed, as well as high levels of proinflammatory cytokines such as interleukin 1 (IL-1), interleukin 6 (IL-6), tumor necrosis factor alpha (TNF α) and C-reactive protein (CRP) [11]. Because of the endothelium damage, patients with DFU show low levels of vascular endothelial growth factor (VEGF), which means poor angiogenesis that contributes to impaired healing. Patients with diabetes also express low levels of nuclear factor erythroid-2-related factor 2 (Nrf2), a molecule that is activated as a response to hypoxia, so it plays a role in the regulation of angiogenesis [12]. Another key role in wound healing is played by macrophages. There are pro-inflammatory macrophages, called M1, and anti-inflammatory macrophages, called M2. M1 cells secrete reactive oxygen species (ROS), IL-1, IL-6, TNF α, and matrix metalloproteinases such as matrix-metalloproteinase 9 (MMP-9) and matrix-metalloproteinase 2 (MMP-2), whereas M2 promotes angiogenesis by secretion of VEGF, tumor growth factor beta (TGF β), platelet-derived growth factor (PDGF), and tissue formation [13]. These characteristics could serve as the foundation for developing medication aimed at modulating macrophage polarization as well as suppressing the production of specific interleukins, therefore inhibiting inflammation and assisting wound healing (Figure 1).

## 3. Conventional Management of DFU

The diabetic foot ulcer is a complex pathology associated with many underlying conditions. The management is often multidisciplinary, involving multiple treatment methods, although none have proven complete effectiveness. The goal of DFU treatment is to allow proper wound healing whilst maintaining the adequate function of the limb [14].

The first step is blood sugar control, avoiding hyperglycemia or hypoglycemia. Many patients with diabetes require pharmacological therapy like antidiabetic medications, statins, antiplatelets, or antibiotics in case of an infection [15]. Standard care for the DFU consists of pressure off-loading, large debridement of tissue, and wound moisture bandages [16].

In recent years, new treatment methods for DFU have developed. One of them is hyperbaric oxygen therapy (HBOT). Because the tissue of the diabetic wound is hypoxic, HBOT delivers oxygen at a higher pressure and it is proven to promote angiogenesis and fasten wound healing [17]. However, a meta-analysis concluded that HBOT had no impact on overall amputation rates or mortality and that adverse reactions were more commonly observed than in the standard care treated patients; therefore, its usage calls for cautions, as well as other clinical trials to assess its efficacy and safety [18].

Another strategy is negative pressure wound therapy (NPWT), which has an increased clinical application, because of its advantages of fast healing and infection control, by reducing secretions and promoting granulation tissue formation. Although research studies on its efficacy are limited, it is generally considered a safe treatment but should be used with caution to prevent wound bleeding [19].

In the last few years, platelet-rich plasma (PRP) therapy has gained more utility in treating diabetic ulcers. PRP is a plasma preparation that has a concentration of platelets 4–5 times higher than whole blood. The blood is centrifugated, and then the platelets are harvested [20]. A meta-analysis of 19 studies found that PRP in DFU promoted wound healing and had serious benefits and a higher wound closure rate compared to the control group who received standard care, while another study on sixty patients concluded that PRP has no better effect in DFU healing than saline wound dressings [21].

Another alternative for treating DFU that is currently under study is skin grafting. Its limitations and uncertainties stem from the invasive procedure required for graft harvesting in autografts, as well as the risk of rejection and immune reactions associated with allografts [22].

## 4. Novel Therapeutic Approaches

Recent studies in biomedical research have introduced innovative treatment strategies that hold promise for improved healing outcomes. In addition to conventional treatments and those previously outlined, the utilization of stem cell-based therapy for the healing of diabetic ulcers has recently gained significant momentum. Many studies throughout the years have examined these cells and characterized their potential for wound healing.

### 4.1. Stem Cell Therapy: Advanced Wound Healing

One of the first studies on embryonic stem cells (ESCs) and wound healing, conducted by Lee et al. in 2011, demonstrated that topical application of ESCs to diabetic mice’s wounds enhanced re-epithelialization, angiogenesis, and ulcer healing [23]. However, the clinical use of ESCs remains limited due to ethical concerns surrounding their extraction and the potential risk of teratoma formation [24].

To overcome these challenges, induced pluripotent stem cells (iPSCs) have been developed by reprogramming adult somatic cells, such as fibroblasts, back into a pluripotent state. These cells exhibit pro-angiogenic properties and have been shown to increase collagen deposition and wound perfusion, therefore assisting the process of wound healing [25].

While both ESCs and IPSCs offer regenerative capabilities, their clinical translation is constrained by safety and ethical considerations. In contrast, mesenchymal stem cells (MSCs), which are multipotent and capable of self-renewal and differentiation into various cell types, have attracted considerable interest for clinical applications [26]. Notably, MSCs express low levels of HLA class I molecules, which reduces the risk of immune rejection [27].

Among MSCs, two types stand out for their accessibility and safety profiles: bone-marrow-derived MSCs (BM MSCs) and adipose-derived MSCs (ASCs). BM MSCs, obtained from the spongy bone tissue of the femur or tibia, aid in immune modulation, vascular proliferation, and tissue regeneration. ASCs, however, have become more extensively studied in wound healing due to several advantages such as minimally invasive collection, obtained in larger quantities through multiple harvests, longer lifespan, shorter doubling time, and greater proliferative potential [28,29,30].

ASCs are isolated from the stromal vascular fraction (SVF) of adipose tissue, a heterogeneous cell mixture that also includes pre-adipocytes, fibroblasts, endothelial progenitors, macrophages, and lymphocytes. While ASCs can be expanded for both allogeneic and autologous therapies, the unprocessed SVF is typically limited to autologous applications [29,31]. SVF injection presented its safety and effectiveness in treating DFU, in a study involving 63 patients with type 2 diabetes mellitus (T2DM), where after 12 months of follow-up, 50 patients maintained complete wound closure without adverse reactions [32]. Bi et al. further explored the roles of SVF and ASCs in skin wound healing and found that both cell populations enhanced fibroblast and endothelial cell functions, regulated gene expression, and promoted skin regeneration. These effects appeared to be mediated through mechanisms such as fibroblast migration and capillary formation, involving the regulation of cell adhesion molecules and cytokine signaling pathways [33].

ASCs are particularly valuable in wound healing due to their high plasticity, ability to differentiate into various cell types, and secretion of key growth factors, including VEGF, fibroblast growth factor-2 (FGF-2), TGF-β 1, insulin growth factor-1 (IGF-1), hepatocyte growth factor (HGF), PDGF, and keratinocyte growth factor (KGF), that promote angiogenesis and tissue regeneration. Their ability to migrate to injury sites, along with their anti-apoptotic, antioxidant, anti-inflammatory, and antibacterial properties, makes them a compelling therapeutic option for treating chronic wounds [30,34,35].

Adipose-derived stem cells (ASCs) have been investigated in multiple studies to evaluate their therapeutic potential and safety in promoting ulcer healing. Various formulations of ASCs present numerous opportunities as active agents or ready-to-use therapeutic materials. In one study, Seung and colleagues utilized human processed lipoaspirate (PLA), injecting it directly into the wound site of patients with chronic diabetic ulcers. Their results were promising: all patients treated with PLA achieved complete wound healing within eight weeks of the initial procedure, with the fastest healing observed by day 17. In contrast, only 62% of patients in the control group—who received topical fibrinogen and thrombin—experienced full healing [36]. Others have administered ASCs intradermally around ulcers in streptozocin-induced diabetic rats, resulting in faster healing than the control group [37]. Xenogeneic ASC sheets from human donors also seem to be effective, as proven in a study by Hamada et al., where the xenografts were transplanted to Zucker Diabetic Fatty (ZDF) rats with large induced ulcers, resulting in enhanced wound healing by increasing the wound closure rate, blood vessel density, and dermal thickness [38].

Since chronic leg ulcers are often not solely attributed to diabetes, adipose-derived regenerative cells (ADRCs) have also been explored as a treatment option for patients with peripheral arterial disease. In this context, isolated ADRCs were injected around the margins of chronic wounds and demonstrated positive therapeutic outcomes [39].

Similarly, transplantation of ASCs into the femoral vein of 18 diabetic mice showed, on day 15 of follow-up, a significant reduction in ulcer size compared to the control group, along with more intense granulation tissue formation and collagen deposition [40].

ASCs’ therapeutic effect is mediated through their secretome, a collection of bioactive molecules secreted into the extracellular space. This includes proteins, growth factors, cytokines, and extracellular vesicles such as exosomes (EXOs). Stem cell therapy challenges make the ASC secretome offer an innovative and potentially safer cell-free alternative [41].

Despite their major potential in wound healing, ASCs face practical and biological limits. Isolation during liposuction reduces cell viability, and local anesthetics can impair their yield and function. Intravenous delivery is inefficient, with most cells failing to reach the wound or dying quickly. Even local delivery struggles with cell survival and integration. Their effectiveness is further affected by patient factors like age, comorbidities, and wound conditions, which impact ASCs proliferation, differentiation, and secretion [42].

These limitations highlight the need for more efficient and targeted approaches. Exosome-based therapies are a promising solution, offering safer and more controlled local delivery of factors or drugs, thus overcoming the challenges of stem cell therapy in diabetic wound healing.

### 4.2. Exosomes—Exploring a New Frontier in Wound Healing

EXOs are small extracellular vesicles produced by most cells, carrying proteins, lipids, deoxyribonucleic acid (DNA), ribonucleic acid (RNA), including micro-RNA, and other signaling molecules that transport cytokines, ECM proteins, heat shock proteins, and drugs involved in various pathophysiological processes. ASCs secrete larger EXOs (ASCs-EXOs), which enhance fibroblast and endothelial cell proliferation and migration, aiding wound healing through anti-inflammatory, angiogenic, and immunosuppressive effects, by secreting growth factors (VEGF, FGF, PDGF, TGF-β1), inhibiting interferon- α (IFN-α) and T lymphocytes, and promoting ECM reconstruction [43,44,45]. The advantages that ASCs-EXOs possess over ASCs alone, such as easier preservation and transport, greater stability, or the ability to cross the blood-brain barrier [45], have made ASCs-EXOs a highly studied topic for innovating the approach to non-healing wounds, especially DFU.

Numerous studies have demonstrated the beneficial effects of ASCs-EXOs in diabetic wound treatment through various mechanisms. Beyond their positive effects mentioned above, EXOs have also been studied for their influence on the macrophages from the adipose tissue. Macrophages possess two phenotypes: M1—pro-inflammatory and M2—pro-healing. In a diabetic environment, M1 macrophages dominate the wound site; therefore, strategies for converting M1 to M2 are needed [46]. Exosomes derived from M2 macrophages (M2-EXOs) are proven to promote wound healing by their anti-inflammatory properties, fibroblast proliferation, secretion of growth factors such as VEGF, and angiogenesis [45]. Xia et al. [47] investigated whether EXOs derived from lean adipose tissue macrophages (EXOs^lean^) could enhance wound healing in diabetic mice. They found that EXOs^lean^, enriched with microRNA (miR-222-3p), promoted the polarization of macrophages from the pro-inflammatory M1 phenotype to the anti-inflammatory M2 phenotype. This modulation led to accelerated wound closure, reduced inflammation, and improved tissue regeneration, highlighting EXOs^lean^ as a promising immunoregulatory treatment for chronic diabetic wounds.

**Figure 2:**
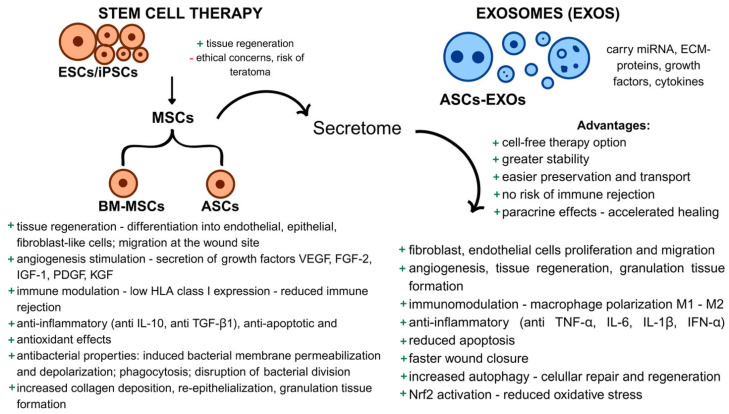
Comparison between stem cell therapy and exosome-based therapy in wound healing. On the left, stem cell therapy involves pluripotent stem cells, such as ESCs (embryonic stem cells) and iPSCs (induced pluripotent stem cells), which have high regenerative potential but are associated with ethical concerns and teratoma risks. Stem cells can differentiate into MSCs (mesenchymal stem cells), including BM-MSCs (bone marrow-derived) and ASCs (adipose-derived stem cells), which mediate healing through paracrine signaling (the secretome), releasing growth factors like VEGF, FGF-2, and PDGF to stimulate angiogenesis, tissue regeneration, and immune modulation. MSCs also exhibit anti-inflammatory, anti-apoptotic, and antibacterial effects, enhance collagen deposition, and promote re-epithelialization. On the right, exosome-based therapy uses ASCs-derived exosomes (ASCs-EXOs) as a cell-free alternative. EXOs carry miRNAs, growth factors, and cytokines, offering advantages such as stability, ease of transport, and absence of immune rejection. They accelerate healing by promoting fibroblast and endothelial cell proliferation, angiogenesis, immune modulation, and reducing oxidative stress. EXOs also have anti-inflammatory effects and enhance tissue regeneration and wound closure through Nrf2 activation and autophagy.

Also, ASCs-EXOs positively impacted diabetic wound healing in vivo by increasing autophagy and accelerating tissue repair by re-epithelialization. This suggests that autophagy played a key role in this context, offering insights into their regulatory mechanism [48]. Another important research finding focused on Nrf2, a transcription factor involved in the cellular response to oxidative stress. EXOs from ASCs overexpressing Nrf2 significantly reduced the ulcerated area in a diabetic rat model and enhanced wound healing compared to the untreated control, with notable improvements in granulation tissue formation, angiogenesis, growth factor expression, and a decrease in inflammation and oxidative stress-related proteins in the wound areas of treated rats [49]; Figure 2.

## 5. Drug-Loaded Scaffolds: A Breakthrough in Chronic Wound Healing

Recent research has focused on developing innovative treatment methods utilizing biomaterials. These advanced approaches aim to revolutionize DFU management by accelerating wound healing, reducing inflammation, and preventing infections and severe complications. Leveraging the unique properties of biomaterials, these treatments offer personalized and effective solutions, paving the way for new advancements in addressing this complex condition.

In the following, we will explore various scaffolds with drug delivery capabilities and carrier potential that demonstrate significant promise in enhancing the healing process of diabetic wounds.

### 5.1. Microfragmented Adipose Tissue (MFAT) in DFU Healing: A Revolutionary Approach for Regeneration and Repair

In recent years, increased attention has been given to developing biological scaffolds that require minimal to no chemical involvement for their preparation, thereby emphasizing the natural properties these scaffolds possess. One such biological material is micro-fragmented adipose tissue (MFAT), widely known for its anti-inflammatory, pro-healing, and drug delivery capabilities, with current applications in treating perianal fistulas, osteoarthritis, and wound healing [50].

Micro-fragmentation is a process that can be performed using various devices specifically designed for this purpose. Lipoaspirate is introduced into the Lipogems^®^ device and initially filtered to reduce cluster size. Mechanical agitation with stainless steel marbles promotes micro-fragmentation of adipose tissue, while a saline counterflow removes blood and residual oil. Once the solution is clarified, the device is inverted, and a secondary filtration further refines the tissue. The resulting MFAT is then aspirated for subsequent analysis [51]. The final product comprises a heterogeneous mixture of stromal progenitor cells, including ASCs, functionally akin to MSCs, as well as pericytes, endothelial cells, and immunoregulatory components such as white blood cells. Altogether, these components constitute the SVF [52].

Enzymatic processing of adipose tissue results in significant loss of its ECM. In contrast, mechanical manipulation and size reduction preserve structural collagens and maintain the native microenvironmental niche, potentially offering advantages over isolated SVF, such as improved cushioning, structural support, and enhanced graft retention and integration post-treatment [53].

ASCs appear to be the most valuable element from MFAT as they are highly attractive for regenerative medicine due to their multipotency, ease of isolation, abundance, suitability for autologous or allogeneic use, pro-angiogenic capacity, and demonstrated anti-inflammatory and immunomodulatory effects, which have prompted extensive preclinical and clinical research [54].

Through analysis of their molecular profile, some authors have suggested that ASCs possess the capacity to suppress the proliferation and invasiveness of glioblastoma cells [55].

In addition to these antitumor properties, ASCs have also been recognized for their potential as drug delivery vehicles. A growing body of research has demonstrated that various therapeutic agents can be incorporated into MSCs, including ASCs, and subsequently released via extracellular vesicles (EVs), offering a promising strategy for targeted and sustained delivery of bioactive compounds in cancer and other disease contexts [56,57,58].

Beyond oncology, the therapeutic versatility of ASCs is further highlighted by their contributions to regenerative medicine. For instance, lipoaspirate, the adipose tissue-derived product from which ASCs are often isolated, possesses multiple benefits for wound healing, including fibroblast and keratinocyte proliferation, an antibacterial effect on *E. coli* and *P. aeruginosa*, expression of growth factors and cytokines, as well as high stability and safety [59].

The therapeutic potential of autologous micro-fragmented adipose tissue (MFAT) has been explored in both case-based and controlled clinical settings, particularly in the context of chronic, non-healing ulcers.

A case report [60] described the application of MFAT prepared using the Lipogems system in a patient with a non-healing ulcer on a prosthesis residual limb. Four weeks following treatment, the ulcer demonstrated marked improvement, accompanied by a significant reduction in pain. While these findings are promising, they are primarily applicable to ulcers of diabetic etiology and should be interpreted with caution.

Complementing this observation, a randomized controlled trial [61] involving 114 patients who had undergone minor amputations due to diabetic foot complications compared MFAT injections prepared with the Lipogems kit to standard care. After six months, the MFAT-treated group showed a significantly higher healing rate (80%) compared to the control group (46%), with no treatment-related adverse events reported. These results underscore the potential of MFAT as a safe and effective adjunct in the management of diabetic foot ulcers.

Also, the combination of ASCs and exendin-4, an anti-diabetic drug, was found to accelerate wound healing and tissue regeneration in diabetic mice [62]. Exendin-4 is a peptide that shares amino acid similarities with the human incretin hormone glucagon-like peptide-1 (GLP-1) and acts as a potent GLP-1 agonist receptor. Due to its resistance to enzymatic degradation, exendin-4 has a prolonged biological effect compared to GLP-1, which has led to its development as a therapeutic agent for T2DM. It was found that this drug possessed anti-inflammatory, anti-cancer, re-epithelializing, angiogenic, neuroprotective, as well as cardiovascular and renal preserving properties [63]. Exendin-4 gene modification and microscaffold encapsulation enhanced the antidiabetic effects of MSCs, boosting their therapeutic potential for diabetes treatment by improving cell survival, insulin sensitivity, glucose tolerance, and reducing liver fat in diabetic mice. This approach promoted sustained MSC survival and function, offering a promising strategy for long-term blood glucose regulation in patients with persistent hyperglycemia [64].

As aforementioned, ASCs and EXOs are two of the crucial components of the innovative treatment for DFU. The downside is that they are difficult to manipulate and stabilize, which has led researchers to find new methods for consolidation, by incorporating them into different scaffolds.

Two clinical studies assessed the injection of adipose tissue mesenchymal stem cells into the limb with ischemia of patients with diabetes, after the tissue was harvested through liposuction [65,66]. The intramuscular injection appears to be safer than intravenous, and the liposuction is a minimally invasive procedure that is well tolerated by the patients. This innovative treatment showed no adverse reactions, demonstrating high safety and feasibility for further use.

In this direction, Ma et al. [67] developed a non-invasive, painless, antimicrobial microneedle patch for delivering the adipose tissue-derived apoptotic vesicles (ApoEVs-AT@MNP) based on HA for infected wounds. Due to this transdermal administration method, the drug is released into the subcutaneous capillaries, facilitating local absorption and wound healing.

A novel application of ASCs involves their aggregation into three-dimensional (3D) spheroids. These ASC spheroids exhibit enhanced regenerative capacity, improved mechanical stability, and a reduced risk of cellular migration due to their structural properties, making them particularly promising for wound healing applications. In a study by Feng et al. [68], both in vitro and in vivo experiments were conducted to evaluate the therapeutic potential of ASC spheroids embedded in an HA matrix. Using a chronic wound model in mice, applied to both irradiated and non-irradiated skin, the researchers administered various cellular products or vehicle controls. Notably, mice treated with HA-embedded ASC spheroids on irradiated ulcers exhibited near-complete wound closure by day 10, whereas wounds in the other treatment groups remained unhealed even at day 18. These findings demonstrate the considerable potential of ASC spheroids in promoting wound closure, angiogenesis, and tissue regeneration.

An innovative approach to the treatment of DFUs was explored in a recent study utilizing 3D printing technology to create an autologous, minimally manipulated homologous adipose tissue (AMHAT) scaffold. Following precise measurement of ulcer dimensions, the customized AMHAT scaffold was printed and directly applied to the wound site. Among ulcers that were previously unresponsive to standard care, 60% (6 out of 10) achieved complete healing within 12 weeks. Notably, five of these ulcers healed by week 6, and one by week 8. The procedure was well tolerated, with no reported adverse reactions or patient discomfort. These findings highlight the potential of 3D-printed AMHAT as a promising therapeutic alternative for refractory DFUs [69].

Despite the promising therapeutic potential of MFAT in regenerative medicine, several limitations must be considered. One primary concern is the heterogeneity of MFAT preparations, which can vary based on donor characteristics, harvesting techniques, and processing protocols, leading to inconsistent clinical outcomes [70]. The lack of standardization in MFAT processing devices and procedures, such as the Lipogems system, also presents challenges in reproducibility and regulatory approval [71]. Furthermore, clinical evidence remains limited, with many existing studies being small-scale, lacking control groups, or showing methodological variability, thus hindering definitive conclusions regarding efficacy [72]. Finally, long-term safety data are sparse, and while MFAT appears well tolerated in the short term, its effects over extended periods remain largely unknown.

The quality and regenerative potential of adipose tissue harvested from diabetic patients may be significantly compromised, posing limitations for autologous applications such as MFAT or ASC-based therapies. Several studies have demonstrated that adipose tissue from individuals with diabetes exhibits altered cellular composition, including a reduction in MSC yield, impaired proliferation and differentiation capacity, and increased cellular senescence [73]. Chronic hyperglycemia and systemic inflammation associated with diabetes contribute to oxidative stress, mitochondrial dysfunction, and epigenetic alterations in ASCs, diminishing their paracrine signaling and angiogenic potential [74].

These findings suggest that while MFAT derived from diabetic patients can still be used, its therapeutic efficacy may be reduced compared to that from non-diabetic sources, and outcomes may be more variable. As such, patient selection, donor tissue screening, and potential preconditioning or enhancement strategies (e.g., hypoxic culture, growth factor supplementation) may be important considerations for improving results in diabetic populations [75].

### 5.2. Hydrogels in Chronic Wound Care

Unlike the traditional inert materials that are currently used for treating ulcers, such as bandages and gauzes, hydrogels ideally should be biocompatible, non-toxic, capable of drug delivery, and able to maintain a moist environment at the site of the injury, while absorbing the wound exudate. Additionally, they should provide mechanical protection, act as a barrier against bacteria and infections, and, most importantly, promote the healing of the ulcer by tissue regeneration [76].

Natural biological hydrogels are ECM-like, 3D polymeric structures that have been widely used and studied in clinical trials under different combinations for chronic and diabetic wound healing [76,77]. By integrating antioxidants and other substances into their structure, hydrogels become a potentially more effective wound dressing that can restore the normal tissue micro-environment and promote healing [77].

#### 5.2.1. Chitosan—Properties, Applications, and Limitations in Diabetic Wounds

Among the oldest and most used biomaterials for wound healing is chitosan. It is a natural polysaccharide, derived from chitin, a compound found in the crustacean exoskeleton [78]. Chitosan has been widely used in the medical field, exhibiting therapeutic potential in the management of gastric ulcers, cancer, genotoxicity, and wound healing [79]. It has moisturizing and hemostatic effects, good biocompatibility and biodegradability, mucoadhesiveness, lack of immunogenicity, and excellent antibacterial properties [80]. Chitosan exhibits anti-inflammatory properties, which are beneficial in therapeutic contexts such as wound healing, bone regeneration, and gastrointestinal treatment. It reduces the expression of pro-inflammatory cytokines, including TNF-α, IL-6, and IL-1β, while increasing the production of anti-inflammatory markers such as IL-10 and TGF-β1 [81].

Additionally, chitosan promotes and serves as a carrier for the release of PDGF and TGF, supports angiogenesis, and facilitates the formation of collagen and ECM proteins [79]. It was demonstrated that chitosan promotes the growth of beneficial bacteria that aid in wound healing while simultaneously protecting the wound from infections. Its effect is primarily directed against *Staphylococcus* spp. and Gram-negative bacteria, such as *E. coli* and *Enterococcus* spp., by binding to the bacterial DNA, thereby inhibiting mRNA and protein synthesis [79,80,82]. Additionally, chitosan disrupts bacterial cell membranes through electrostatic interactions, increases membrane permeability, and chelates essential ions, all of which contribute to bacterial cell death while supporting a favorable environment for tissue regeneration [83].

Chitosan is a versatile biomaterial that can be used in different forms for wound healing, such as scaffolds, hydrogels, or sponges [79]. Phosphorylated chitosan (PC), a hydrosoluble form of chitosan with enhanced antioxidant activity and metal chelation capacity was evaluated in a study involving 15 Wistar rats with streptozotocin-induced diabetic wounds. By day 14, the wounds were evaluated. In the PC-treated group, greater collagen deposition, a thicker epithelial layer, increased angiogenesis, and a higher number of fibroblasts were observed than in the control group, indicating accelerated wound healing [84].

Ullah et al. [85] developed a chitosan-PVA (polyvinyl alcohol) patch enriched with calcium peroxide (CPO) to combat tissue hypoxia related to diabetic ulcers. The study was conducted on Wistar rats with induced diabetic wounds, monitored over a period of 14 days. The results showed an increase in the number of fibroblasts and endothelial cells at the wound site, along with blood vessel formation and tissue regeneration in the treated group compared to the control group. Additionally, antibacterial and antifungal effects were observed at the site of ulceration.

To further enhance the effects of chitosan, as well as to improve the stability and release of exosomes, Shang et al. [86] designed a hydrogel based on carboxymethyl chitosan (CMCS), loaded with MSC-derived exosomes (MSC-EXOs), bioactive glass (BG), and titanium dioxide (TiO_2_) to treat acute and chronic diabetic wounds. In vitro, the hydrogel demonstrated excellent biocompatibility, promoting endothelial cell adhesion and proliferation, while exhibiting anti-inflammatory, angiogenic, and antibacterial effects. In vivo, using rat models with full-thickness skin defects, diabetic wounds, and burns, the hydrogel significantly accelerated wound healing compared to controls, with increased collagen deposition, enhanced angiogenesis, and upregulated expression of anti-inflammatory markers.

Despite its advantages and great promise for healing diabetic wounds, chitosan presents mechanical and immunological limitations that can affect its clinical performance. One major weakness is its limited solubility at physiological pH, as chitosan dissolves only in acidic environments. Additionally, commercial chitosan varies in molecular weight and degree of deacetylation depending on its source, leading to inconsistent biological effects and challenges in standardization. Chitosan hydrogels often suffer from poor mechanical strength, rapid degradation, and limited adhesion to tissues, factors that can interfere with their effectiveness in wound repair applications [87]. Addressing these limitations typically involves chemical modifications, combination with other polymers, or incorporating inorganic materials to improve chitosan’s solubility, mechanical properties, and biological performance.

In addition to mechanical limitations, chitosan can exert pro-inflammatory effects under certain conditions, such as when used in powder form, at high concentrations, with low molecular weight or partially deacetylated chitosan. These forms can activate immune cells and stimulate pathways like NF-κB, resulting in the release of inflammatory cytokines, such as IL-1β. While this pro-inflammatory activity can be useful in applications like vaccine delivery or cancer therapy, it may be harmful in tissue repair [81]. Therefore, the immunological response to chitosan must be adjusted through formulation and material design to address the therapeutic goal.

#### 5.2.2. Alginate—Therapeutic Use and Challenges in Diabetic Ulcers

Alginate, a natural polysaccharide extracted from brown algae, is one of the most commonly used hydrogels in wound healing due to its excellent biocompatibility, low cost, non-toxic profile, hydrophilic nature, and potential as a drug or cell delivery carrier [76,78]. While effective, alginate presents several limitations for independent application, particularly in DFUs.

One major limitation is its poor mechanical strength. Alginate has a soft nature and is prone to premature degradation, making it unsuitable for long-term structural support. To improve its scaffold properties, alginate is often combined with other materials like chitosan, collagen, or gelatin. While alginate is biocompatible, it lacks bioactivity that promotes healing and is frequently loaded with different bioactive molecules or therapeutic agents to enhance its effect [88].

In the context of DFU, alginate formulations have demonstrated significant therapeutic potential. For instance, sodium alginate hydrogels loaded with Benlysta, an immunomodulatory drug, have been shown to stimulate fibroblast and epidermal cell proliferation, enhancing tissue regeneration and accelerating wound closure [89].

Calcium alginate is an alginate hydrogel variant with good compatibility and no toxicity that has proven effective in wound healing. In diabetic rat models, it significantly increased collagen density, modulated the collagen I/III ratio, enhanced fibroblast activity, and reduced inflammation while maintaining a moist wound environment, with a higher wound closure rate than the control group [90].

To further optimize alginate’s therapeutic potential, researchers have developed composite hydrogels by integrating other bioactive components. Yan et al. introduced a hybrid hydrogel system incorporating microgel particles synthesized through the coordination of zinc ions with carboxymethyl chitosan (CMCS) and aldehyde hyaluronic acid (A-HA), which serve as primary crosslinkers, providing a flexible network structure. In vitro studies demonstrated that these hydrogels support cell infiltration and proliferation, while in vivo experiments using diabetic mouse models showed accelerated wound closure, enhanced collagen deposition, and improved tissue regeneration compared to the control group [91]. Similarly, Shah et al. [92] developed a chondroitin sulfate grafted alginate (CS-Alg-g-PF127) hydrogel that could promote wound healing in diabetic rat models. In vitro analyses confirmed the hydrogel’s biocompatibility, antioxidant, and antibacterial properties, while in vivo studies in diabetic rat models demonstrated significant improvements in wound healing, including increased re-epithelialization, angiogenesis, and collagen deposition, while inhibiting inflammation.

A 2023 study by Sheng et al. [93] investigated the therapeutic potential of sodium alginate/gelatin (Gel-Al) hydrogels loaded with ASCs in promoting wound healing in streptozocin-induced diabetic rats. The Gel-Al + ASC group exhibited faster wound closure, enhanced granulation tissue formation, increased collagen deposition, and better re-epithelialization compared to controls. There was a notable upregulation of angiogenic factors such as VEGF, PDGF, and EGF and the endothelial marker CD31. The treatment also modulated inflammation by decreasing IL-6 and IL-1β and increasing anti-inflammatory cytokines IL-10, IL-4, IL-13, and TGF-β1, along with M2 macrophage polarization. These findings suggest that Gel-Al hydrogels loaded with ASCs provide a favorable microenvironment for stem cell viability and function, leading to improved healing outcomes in diabetic wounds.

#### 5.2.3. Collagen—Use and Limitations in Tissue Repair

Collagen is a key component of the ECM, and its potential in wound healing derives from its biocompatibility, biodegradability, and ability to support cellular processes involved in tissue regeneration. As a natural scaffold, collagen provides structural support, promotes cell adhesion, and encourages cellular proliferation, migration, and differentiation, all of which are crucial for tissue repair. Additionally, collagen’s ability to maintain a moist wound environment and its hemostatic properties help control bleeding and accelerate healing. However, despite these advantages, collagen presents several limitations in wound healing applications. Its mechanical strength is relatively low, and it degrades rapidly, particularly in chronic wounds such as diabetic ulcers, where high protease activity can compromise its stability. Furthermore, collagen derived from animal sources may exhibit variability in composition, leading to inconsistent biological responses and potential immunogenicity [94]. A multicenter randomized controlled trial by Veves et al. [95] compared Promogran, a collagen/oxidized cellulose dressing, with standard moistened gauze in 276 patients with DFU. The Promogran-treated group showed faster wound closure and reduced ulcer size compared to conventional dressings, with no side effects. This study supports the clinical efficacy of collagen in treating diabetic ulcers, although its performance can be enhanced with modifications such as crosslinking or combining it with other materials to overcome its limitations.

### 5.3. Hyaluronic Acid: Promoting Rapid Recovery and Efficient Drug Delivery for DFU Healing

HA is a natural glycosaminoglycan and a major ECM component that is found in the human body, especially abundant in the skin, joints, and connective tissues. Due to its remarkable water-retention capability, HA is vital in maintaining skin hydration, elasticity, and tissue integrity. These properties, together with its biocompatibility and viscoelasticity, make HA a commonly used biomaterial in various fields of application, such as cosmetology, orthopedics, or tissue engineering. In the context of DFU, HA emerges not only as a structural scaffold but also as a multifunctional therapeutic agent [96,97].

Particularly fascinating is the role of ultra-high molecular weight hyaluronic acid (UHMW-HA), which has demonstrated superior anti-inflammatory, antioxidant, and tissue-protective properties compared to standard HA due to its exceptional molecular size and viscoelasticity. In species like the naked mole rat, UHMW-HA contributes to cancer resistance and longevity by preserving ECM integrity and suppressing chronic inflammation, mechanisms that translate into improved chronic wound healing. Unlike standard HA, which can fragment and promote inflammation under pathological conditions, UHMW-HA maintains stability and creates a regenerative micro-environment that reduces immune cell infiltration and oxidative stress [71].

Native HA is rapidly degraded in chronic wound environments by hyaluronidases and oxidative stress, which limits its therapeutic effect. To overcome this, HA is chemically modified through crosslinking (e.g., with aldehydes or methacrylation) to form hydrogels with increased stability, prolonged bioactivity, and controlled drug release [98]

In recent years, HA has been extensively studied for its potential in promoting wound healing, both as an independent agent and as a scaffold for delivering bioactive substances to enhance the repair processes. Studies have demonstrated that HA can support tissue regeneration by facilitating the localized release of substances that promote angiogenesis, cell proliferation, and collagen deposition [99].

For instance, De Angelis et al. [100] evaluated the combination between HA and PRP administration in chronic ulcers. The results showed that this dual approach improved granulation tissue formation, prevented infection, and reduced swelling and pain. HA acted as a scaffold that maintained hydration at the wound site, while PRP activated cell proliferation, thereby improving wound healing.

In another study, HA was used as a matrix for dopamine (DA), an anti-inflammatory and antioxidant substance from mussels, creating a hydrogel that had a positive effect on diabetic wound healing in mice. Not only did it promote angiogenesis and tissue repair, but it also decreased inflammation by changing the polarization of macrophages, proved by a high M2 pro-healing macrophage ratio [101]. Similar findings were determined by Wang et al. [102] when developing a collagen HA scaffold primed with pDA (polydopamine) and EGF (epidermal growth factor) for treating rat diabetic ulcers. CHS-PDA scaffold accelerated cell proliferation and the transformation of M1 macrophages into M2 macrophages, thereby inhibiting inflammation and promoting re-epithelization of the damaged tissue.

HA hydrogels have also been used to deliver paeoniflorin (PF), a compound extracted from *Paeonia lactiflora* with anti-inflammatory properties through modulating macrophage polarization and secretion of pro-healing cytokines such as TGF-beta and IL-10, hence promoting diabetic wound healing [103].

Given the high risk of infection in DFU patients, HA-based hydrogels have been engineered to deliver antibacterial and anti-inflammatory compounds. Zhu et al. [104] designed one such system, Met@-CuPDA NPs/HG, a hydrogel loaded with copper (Cu^2+^) polydopamine nanoparticles and metformin, an anti-diabetic drug. Cu^2+^ proved to be an excellent antibacterial and angiogenetic element, while PDA and metformin had an inhibiting effect on ROS and inflammation, ultimately promoting diabetic wound healing. Multiple studies have demonstrated the potential of metformin in treating chronic or diabetic wounds, not only through its oral antidiabetic administration but also through topical application [105], by increasing collagen production at the wound site and inhibiting MMP 2 and MMP 9 production in a diabetic rat model. Supporting this statement, a spray containing metformin and zinc [106] and a hydrogel loaded with EXOs and metformin [107] have been developed for topical application, both showing great benefits for wound healing in a mouse model, promoting angiogenesis, collagen formation, while also having an anti-inflammatory, antioxidant, and antibacterial effect.

Curcumin has proven to be an antibacterial, anti-inflammatory, and antioxidant compound found in turmeric that has been shown to possess diabetic wound healing properties [108]. The downside is that it is hydrophobic, which means it has to be combined with a hydrophilic substance, like HA. This combination has exhibited great potential for wound healing, by increasing re-epithelization, granulation tissue formation, and collagen production [108]. In a more complex manner, curcumin was loaded along with EGF into an HA and chitosan hydrogel to study its therapeutic effect [109]. This study offered great results regarding this interaction in a diabetic mouse model. Curcumin was released in the early stage of healing, providing anti-inflammatory and antioxidant effects, while EGF was released gradually to support ECM formation, angiogenesis, and tissue regeneration [109].

Despite these advantages, HA therapy faces limitations. Standard HA lacks mechanical strength and degrades rapidly in inflammatory environments, requiring chemical modification for stability. UHMW-HA is expensive and difficult to produce at scale. Variability between production batches, limited long-term stability, and complex requirements for chemically modified HA formulations interfere with consistent clinical application [110].

**Figure 3:**
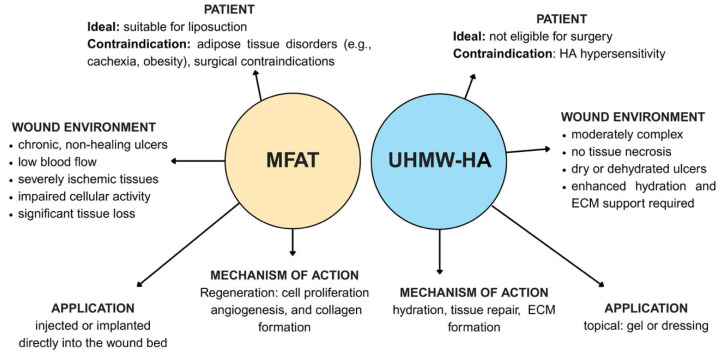
Comparative overview of MFAT (micro-fragmented adipose tissue) and UHMW-HA (ultra-high molecular weight hyaluronic acid) as therapeutic options for wound healing. The figure contrasts the ideal patient profiles, wound environments, mechanisms of action, and methods of application for each material. MFAT is best suited for patients who are eligible for liposuction, with contraindications including adipose tissue disorders such as cachexia or obesity, and any surgical contraindications. It is indicated for chronic, non-healing ulcers in ischemic and low-perfusion tissues, when conventional therapies have failed, especially with impaired cellular activity and significant tissue loss. MFAT is applied by direct injection or implantation into the wound bed and promotes regeneration through cell proliferation, angiogenesis, and collagen formation. In contrast, UHMW-HA is appropriate for patients who are not candidates for surgery, with contraindications including hypersensitivity to HA or specific components of the formulation. It is recommended for wounds that are moderately complex, without tissue necrosis, and characterized by dryness or dehydration, where the tissue is not severely ischemic but requires enhanced hydration and ECM support. UHMW-HA is administered topically, either as a gel or dressing, and contributes to wound healing by enhancing hydration, supporting tissue repair, and facilitating ECM formation.

Multiple studies have highlighted the diverse applications of HA, including its importance and safety usage as a scaffold capable of different drug delivery, positioning it as a key component in the effective treatment of diabetic wounds. A comparison of each of the above scaffolds relating to effectiveness in the treatment of DFU is shown in Table 1 below, and a diagram contrasting MFAT with HA shown as Figure 3.

## 6. Conclusions and Future Perspectives

**Figure 4:**
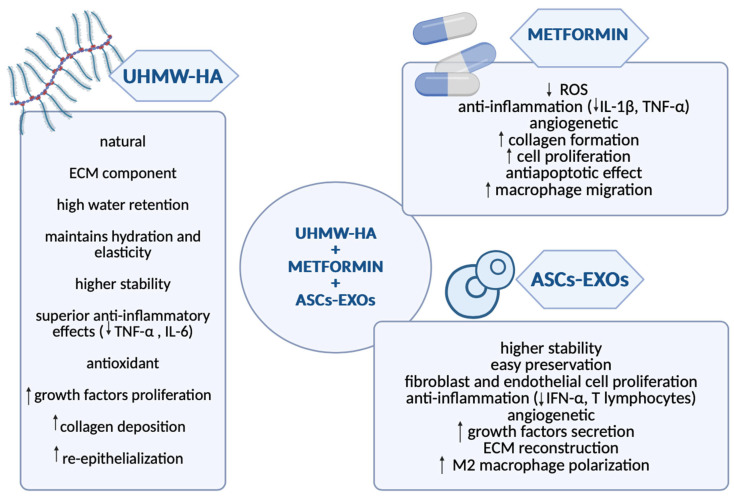
This figure presents a personal approach for a potential future treatment option for diabetic foot ulcers (DFU) using a combination of ultra-high molecular weight hyaluronic acid (UHMW-HA), metformin, and adipose-derived stem cell exosomes (ASCs-EXOs) to enhance wound healing through synergistic mechanisms. UHMW-HA offers superior stability compared to standard HA, promotes hydration, and supports cell migration, collagen deposition, and re-epithelialization, while also reducing inflammation (↓ TNF-α, IL-6) and providing antioxidant effects. Metformin helps reduce reactive oxygen species (ROS), improves insulin sensitivity, stimulates angiogenesis, and promotes collagen formation. ASCs-EXOs contribute to fibroblast and endothelial cell proliferation, immune modulation by promoting M2 macrophage polarization, and extracellular matrix (ECM) reconstruction, shifting the immune response toward tissue regeneration.

DFU represents a pathology driven by complex pathophysiological mechanisms, demanding innovative therapeutic approaches to enhance both ulcer healing and, simultaneously, the patient’s quality of life. The development of advanced scaffolds has become central to improving outcomes in DFU therapy, offering structural and bioactive support in tissue regeneration. Natural materials such as chitosan, alginate, collagen, HA, and MFAT have demonstrated high biocompatibility, bioactivity, and the ability to modulate inflammation, stimulate angiogenesis, and enhance cellular recruitment. Among these, UHMW-HA stands out for its superior viscoelasticity and capacity to serve as a drug delivery support. Increasingly, the combination of these scaffolds with regenerative agents, such as ASCs or EXOs, and pharmacological modulators like metformin offers a promising direction for more targeted and effective therapies, as the example shown in Figure 4 indicates.

Future research should focus on designing composite scaffolds with adjustable mechanical and degradation profiles, incorporating delivery systems, and validating their efficacy in clinically relevant models. The integration of bioactive scaffolds with cell-free therapies presents a promising strategy for personalized and regenerative treatment of chronic DFUs, an area of interest that requires further in-depth research.

## Figures and Tables

**Table 1:** Comparison of the advantages and disadvantages of using MFAT, chitosan, alginate, HA, and collagen in the treatment of DFU.

Natural Scaffolds	Advantages	Disadvantages
**MFAT**	- a natural source of ASCs/SVF - structural support - biocompatible - anti-inflammatory and pro-healing - antibacterial effect - promotes secretion of regenerative factors (e.g., VEGF, TGF-β) - no adverse effects - flexible delivery methods (injection, microneedles, etc.) - enhanced drug delivery	- requires liposuction (minimally invasive procedure) - fragile - need for complex equipment (Lipogems) - may yield insufficient tissue - inflammatory phenotype in diabetics - harder to standardize
**Chitosan**	- biodegradable, biocompatible - mucoadhesive - non-toxic - non-immunogenic - various application forms (hydrogels, sponges, etc. - moisture retention - hemostatic - antibacterial: against *Staphylococcus* spp., *E. coli*, *Enterococcus* spp. - promotes growth of beneficial bacterial - anti-inflammatory: ↓ TNF-α, IL-6, IL-1β; ↑ IL-10, TGF-β1 - promotes collagen formation, angiogenesis, ECM protein deposition - carrier for growth factors, EXOs, and other drugs	- poor solubility at physiological pH - variable quality - weak mechanical strength - rapid degradation - may induce inflammation in some forms (powder, partially deacetylated, etc.)
**Alginate**	- biodegradable, biocompatible - affordable - non-toxic - moisture retention - hydrophilic - supports fibroblast activity - drug delivery - effective in composites	- weak mechanical strength - rapid degradation - lack of bioactivity when used alone - needs to be combined with other agents
**HA**	- ECM mimic - strong hydration - maintains elasticity - high water retention capacity - anti-inflammatory (↑ IL-10, TGF-β1; M2 macrophage polarization) - promotes angiogenesis, re-epithelization, collagen deposition - effective drug and cell delivery scaffold - UHMW-HA: higher stability; superior anti-inflammatory and tissue-protective effects; maintains ECM integrity	- rapid degradation (standard HA) - UHMW-HA is costly and less available - requires reinforcement for structural applications
**Collagen**	- biocompatible - biodegradable - moisture retention - hemostatic - promotes angiogenesis, cell adhesion	- rapid degradation (especially in diabetic wounds) - immunogenicity risk - weak mechanical strength - needs to be combined with other agents

## Data Availability

No new data were created.

## Abbreviations

The following abbreviations are used in this manuscript:

DM	diabetes mellitus
DFU	diabetic foot ulcer
VSMC	vascular smooth muscle cell
AGEs	advanced glycation end products
PARP	poly-ADP ribose polymerase
COX-2	cyclooxygenase-2
ECM	extracellular matrix
IL-1	interleukin- 1
IL-6	interleukin-6
TNF α	tumor necrosis factor alpha
CRP	C-reactive protein
VEGF	vascular endothelial growth factor
Nrf2	nuclear factor erythroid-2-related factor 2
ROS	reactive oxygen species
MMP 2	metalloproteinase 2
MMP 9	metalloproteinase 9
TGF β	tumor growth factor beta
PDGF	platelet-derived growth factor
HBOT	hyperbaric oxygen therapy
NPWT	negative pressure wound therapy
PRP	platelet-rich plasma
ESCs	embryonic stem cells
iPSCs	induced pluripotent stem cells
MSCs	mesenchymal stem cells
BM-MSCs	bone-marrow-derived MSCs
ASCs	adipose-derived MSCs
SVF	stromal vascular fraction
T2DM	type 2 diabetes mellitus
FGF-2	fibroblast growth factor-2
IGF-1	insulin growth factor-1
HGF	hepatocyte growth factor
KGF	keratinocyte growth factor
EXOs	exosomes
DNA	deoxyribonucleic acid
RNA	ribonucleic acid
miRNA	micro ribonucleic acid
IFN-α	interferon alpha
MFAT	micro-fragmented adipose tissue
GLP-1	glucagon-like peptide-1
HA	hyaluronic acid
UHMWA-HA	ultra-high molecular weight hyaluronic acid
pDA	polydopamine
EGF	epidermal growth factor
IL-10	interleukin 10
