# Correction: Moldovan et al. Multiplexing 3D Natural Scaffolds to Optimize the Repair and Regeneration of Chronic Diabetic Wounds. *Gels* 2025, *11*, 430

**DOI:** 10.3390/gels11070549

**Published:** 2025-07-16

**Authors:** Cezara-Anca-Denisa Moldovan, Alex-Adrian Salagean, Mark Slevin

**Affiliations:** 1 Doctoral School of Medicine and Pharmacy, George Emil Palade University of Medicine, Pharmacy, Science, and Technology of Târgu Mureș, 540142 Târgu Mureș, Romania; moldovan.cezara@yahoo.com; 2 Department of Histology, George Emil Palade University of Medicine, Pharmacy, Science, and Technology of Târgu Mureș, 540142 Târgu Mureș, Romania; alex-adrian.salagean@umfst.ro; 3 Center for Advanced Medical and Pharmaceutical Research, George Emil Palade University of Medicine, Pharmacy, Science, and Technology of Târgu Mureș, 540142 Târgu Mureș, Romania

## Error in Affiliation

In the original publication [1], there was an error regarding the affiliation for Cezara-Anca-Denisa Moldovan. The affiliation should be removed: “Department of Dermatology, George Emil Palade University of Medicine, Pharmacy, Science, and Technology of Târgu Mureș, 540142 Târgu Mureș, Romania; moldovan.cezara@yahoo.com”. The authors state that they would like to proceed with the removal of the affiliation due to a misunderstanding regarding their association with the Dermatology Department within the University. This correction was approved by the Academic Editor. The original publication has also been updated.
